# The Treatment of Fractures from a Common-Sense Point of View

**Published:** 1907-09-14

**Authors:** 


### Points in Surgery.

### THE TREATMENT OF FRACTURES FROM A COMMON-SENSE POINT OF VIEW.

### IV. THE VALUE OF MASSAGE AND PASSIVE MOVEMENT.

The real difficulty in the treatment of a fracture lies not so much in reducing the deformity and obtaining sound union, though these may be difficult enough, but in restoring to the patient a useful limb; and the greatest trouble is often experienced when the patient begins to get up and about. Why is this? Because the muscles of a limb which has been kept at rest in splints until union has occurred, a period usually of four to six weeks, have undergone atrophy from disuse and from the hæmorrhage which took place in them at the time of the injury. Moreover, when union does occur, the soft structures in the neighbourhood of the fracture become bound down to the shaft of the bone, so that if the site of fracture is near a joint, movements in that joint are rendered difficult or impossible. Further, if the lesion is in the neighbourhood of a joint, and occasionally also when it is at some distance, the retention of the limb in splints for several weeks will lead to the formation of adhesions in the joint. Any joint, even a quite healthy one, would in time develop adhesions in its synovial cavity if kept completely at rest for a sufficiently long period. It is to avoid these unpleasant consequences that surgeons to-day make much freer and earlier use of massage and passive movement in the treatment of fractures than was formerly the case. Some continental surgeons are so convinced of the wonderful results of this method that they would have us dispense almost entirely with retentive apparatus. This is, of course, driving the case to extremes; but Sir William Bennett, who has devoted special attention to the method, advocates its use in conjunction with splints at a very early date in the treatment of fractures. He claims many advantages for it. It is the most efficient method of combating the muscular spasm from which patients not infrequently suffer even after the fragments have been properly set. Gentle rubbing movements along the limb with the flat of the hand for ten to fifteen minutes will often suffice to soothe the patient when no other treatment short of chloroform or morphia will have any effect. It produces rapid absorption of the effused blood, and prevents disuse atrophy of the muscles by keeping up their tone, and thus preserves the normal nutrition of the limb. Further, it prevents the formation of adhesions either in the joints or in the immediate neighbourhood of the fracture; and if the formation of adhesions can be prevented, the pain and stiffness will be avoided when the patient begins to get about. Stiffness is, of course, directly due to adhesions in the joint or round the fracture. In the case of fractures of the tibia and fibula the patient not infrequently has very severe pain down the calf and along the sole of the foot when he tries to walk. As Sir William Bennett has shown, this is due to implication of the posterior tibial nerve in the adhesions round the fracture. Early massage by preventing the formation of the adhesions will prevent the onset of this pain. And, lastly, massage and passive movement will shorten the final stages of convalescence so that the length of time between the patients getting up and full recovery of the use of the limb should be reduced to a few weeks at the outside, instead of some months, as happens when a fracture is merely treated by retentive apparatus.

It will be more simple in explaining the technique of the manipulations to take a single example—*e.g.*, a fracture of the tibia and fibula; the method can be modified according to the anatomy of the part, so as to be applicable to almost any variety of fracture. It is essential, in the first place, that the fragments be set in apposition with the greatest possible care. The limb should be put on a back splint and two side splints, fastened with webbing and a bandage, so that the site of the fracture can be easily exposed. The first manipulation, which consists of gentle rubbing of the limb upwards with the flat of the hand, may be commenced at once. It should be done for ten minutes twice daily. There is no objection to passing the hand over the actual site of the fracture in this movement if it be done smoothly and uniformly. On the third day passive movement of the muscles and tendons round the ankle-joint should be begun. At first the toes should be gently extended while the leg lies on the back splint to which it is attached by a bandage round the ankle. It is quite easy to perform this movement carefully and gently, so that there is no material movement between the fractured ends of the bone. At the end of the first week the bandage round the ankle may be taken off, and the whole foot moved at the ankle-joint with one hand, while the other steadies the limb on the splint at the site of fracture. Four or five minutes of passive movement daily is sufficient. At the end of the second week attention may be directed to the knee. The leg is gradually bent with one hand under the knee-joint, and the other steadying the foot, the site of the fracture being protected with short splints while this manipulation is in progress. This is, however, the least important movement, since the stiffness nearly always occurs at the joint below the fracture, and no risk should be run if there is the least doubt as to whether the union is strong enough to stand the strain of this movement. From the third week onwards splints may be dispensed with, and daily massage and passive movement continued until the bone is strong enough to stand the patient's weight. After this complete use of the limb will be recovered in a surprisingly short space of time.

